# Diffuse Large B-cell Lymphoma Presenting as Bilateral Renal Masses: Successful Treatment with Dose-adjusted REPOCH (Rituximab, Etoposide, Prednisone, Vincristine, Cyclophosphamide, Doxorubicin) Chemotherapy Regimen

**DOI:** 10.7759/cureus.3814

**Published:** 2019-01-02

**Authors:** Anahat Kaur, Omar Abughanimeh, Yousaf Zafar, Timothy Pluard

**Affiliations:** 1 Internal Medicine, University of Missouri-Kansas City School of Medicine, Kansas City, USA; 2 Hematology and Oncology, Saint Luke's Hospital, Kansas City, USA

**Keywords:** dlbl, renal, da-repoch

## Abstract

Diffuse large B-cell lymphoma (DLBL) is an aggressive type of non-Hodgkin lymphoma (NHL). Renal involvement in NHL is not uncommon in advanced stages; however, it is rare to have kidneys affected early in the course of the disease. Usual chemotherapy regimen for DLBL is rituximab, cyclophosphamide, hydroxydaunorubicin, oncovin and prednisone (R-CHOP). This is a case of a 50-year-old female diagnosed with DLBL who presented with bilateral renal involvement at disease onset and also underwent complete remission after six cycles of dose-adjusted rituximab, etoposide, prednisone, vincristine, cyclophosphamide, doxorubicin (DA-REPOCH). Limited data exist on outcomes of patients with DLBL and renal disease who are treated with high-intensity regimes such as DA-REPOCH. It would be worth looking further into outcomes of DLBL patients especially with renal involvement on DA-REPOCH. Multicenter trials are required to demonstrate which of the two chemotherapy regimens (R-CHOP vs. DA-REPOCH) have better progression-free survival in this particular subset of patients.

## Introduction

Lymphomas are malignant neoplasms of lymphoid cells. Hodgkin and non-Hodgkin are two major types of lymphomas. Renal involvement in non-Hodgkin lymphoma (NHL) is not uncommon in advanced stages; however, it is rare to have kidneys affected early on in the course of the disease or at presentation. Herein we report a case of a 50-year-old female who presented with flank pain. Bilateral renal masses were seen on imaging and renal biopsy was consistent with diffuse large B-cell lymphoma (DLBL). On review of prior imaging studies done seven months before admission, no masses were detected.

## Case presentation

**Figure 1:**
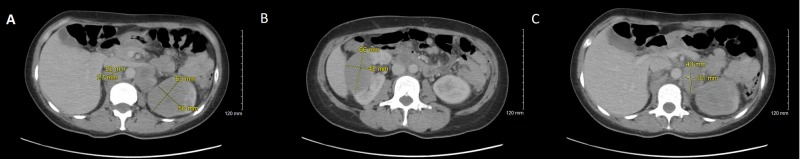
Computed tomography (CT) scan of the abdomen and pelvis A) left renal mass measuring 6.3 x 5 cm. B) right renal mass measuring 6.6 x 4.2 cm. C) left para-aortic lymph node measuring 4.3 x 3.1 cm.

**Figure 2:**
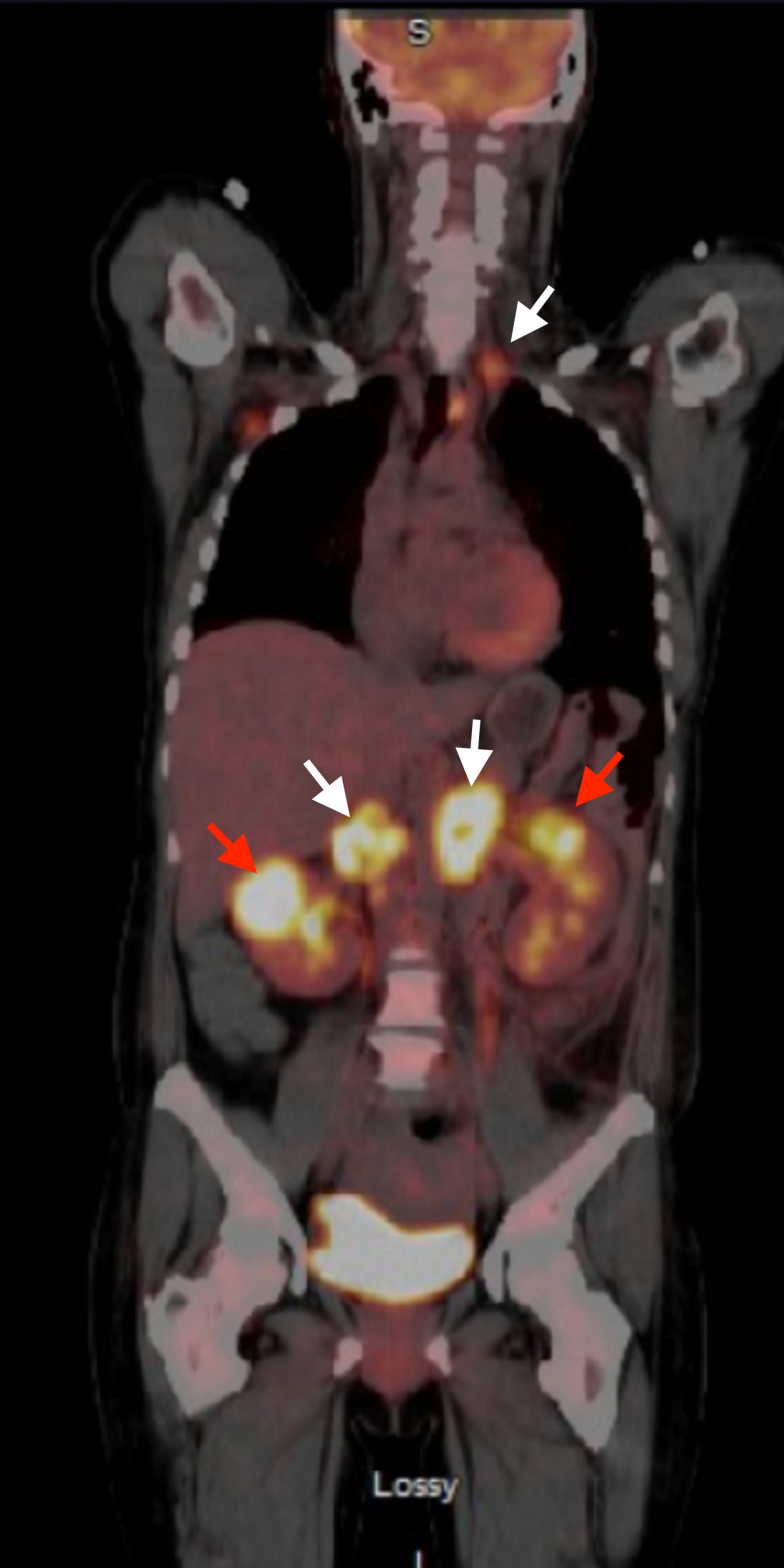
Positron emission tomography/computed tomography (PET/CT) on presentation Left supraclavicular and retroperitoneal lymphadenopathy (white arrows) with hypermetabolic bilateral renal masses (red arrows).

**Figure 3:**
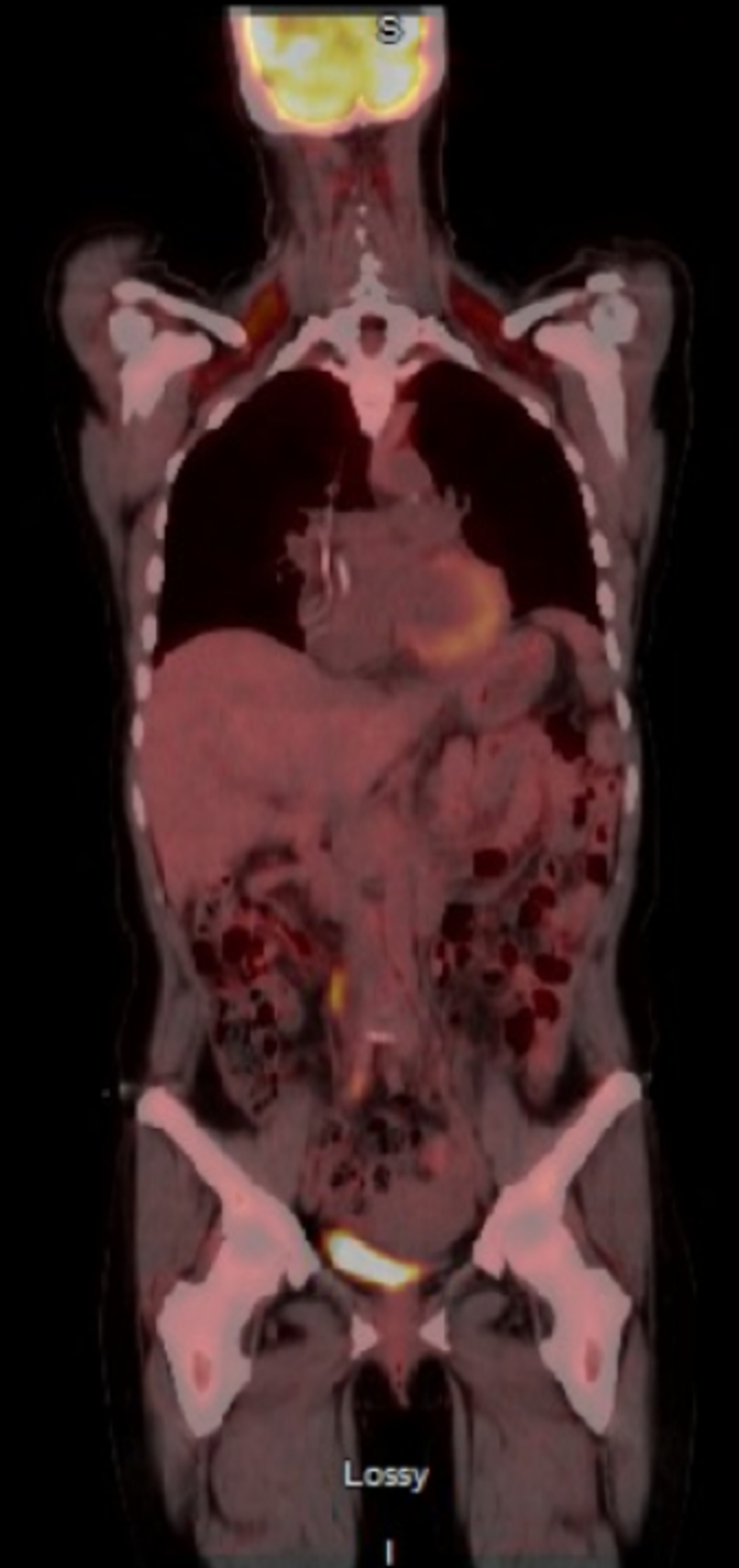
Post-treatment positron emission tomography/computed tomography (PET/CT) No fluorodeoxyglucose (FDG) uptake noted.

A 50-year-old female with a past medical history significant for idiopathic thrombocytopenic purpura (ITP) presented with chief complaint of back pain for three weeks. Prior to her presentation, the patient was undergoing treatment for ITP wherein she had received four doses of weekly rituximab and recently completed a prednisone taper. A computed tomography (CT) scan of the abdomen and pelvis showed bilateral renal masses (6.6 x 4.2 cm on the right, 6.3 x 5 cm on the left) with upper para-aortic and right retro-crural lymphadenopathy (Figure 1). Upon chart review, it was noted that the abdominal ultrasound done seven months prior to admission (for thrombocytopenia workup) was negative for renal masses. She was admitted for evaluation; laboratory workup showed white blood cell count 10.55 TH/uL, hemoglobin 12.3 g/dL, and platelet count 113 TH/uL. Her kidney function and liver function tests were normal. Lactate dehydrogenase was elevated at 763 IU/L (range 313-618). A CT guided biopsy of the left renal mass showed DLBL (Epstein-Barr virus (EBV) negative, fluorescent in situ hybridization (FISH) negative for MYC rearrangement, but 71% of interphase cells showed three copies of an intact MYC (8q24.1), 65% positive for rearrangement of BCL6, no BCL2 fusion). Bone marrow biopsy and flow cytometry were negative. Positron emission tomography (PET)/CT showed left supraclavicular and retroperitoneal lymphadenopathy (standardized uptake values (SUV) 10.8 and 15.7 respectively) with hypermetabolic bilateral renal masses (SUV 15.3 and 17.5 on right and left respectively) (Figure 2). Lumbar puncture cytology was negative. Given these findings, she was staged IVB and received intrathecal methotrexate for central nervous system (CNS) disease prevention. The next day, the patient was started on dose-adjusted rituximab, etoposide, prednisone, vincristine, cyclophosphamide, doxorubicin (DA-REPOCH) chemotherapy regimen. She completed six cycles of DA-REPOCH and intrathecal methotrexate with no evidence of disease on repeat imaging (Figure 3). She continues to follow up with oncology clinic for observation and is in complete remission at one year.

## Discussion

Lymphomas are malignant neoplasms derived from lymphoid cells [1]. Lymphomas are classified into Hodgkin’s lymphoma (HL) and NHL with each one classified further into more than 30 subtypes [2]. DLBL is an aggressive type of NHL and is considered the most common type. The usual presentation of DLBL is a symptomatic enlarged lymph node, 40% cases have stage IV disease on presentation, and 30% of the patients have B symptoms (fever, weight loss, malaise) [2]. Around 40% of DLBL has extra nodal involvement, most commonly in the gastrointestinal tract (36%), ear, nose, throat (20%) and bone marrow (14%) [3].

Although 50% of NHL has renal involvement seen on autopsies, less than 1% is clinically detected in living patients [4]. Yunus et al. studied 901 patients with NHL and found that only 2.1% had renal involvement [5]. Villa et al. performed a retrospective chart review on 2656 patients from January 1982 to December 2008 who were diagnosed with DLBL and renal involvement; the study showed that only 55 patients (2%) had renal involvement at the time of diagnosis. Out of these 55 patients, 50 (91%) had stage IV disease. The study concluded that renal involvement is consistent with extensive nodal or extra nodal disease [6].

Renal involvement in NHL usually occurs in the form of secondary renal lymphoma where there is extensive nodal disease associated with renal involvement. On the other hand, when renal involvement presents with no or minimal lymph nodes, primary renal lymphoma (PRL) is diagnosed [1-2,5,7]. PRL accounts for only 0.7% of all extra-nodal lymphomas and <1% of renal masses [1]. The origin of PRL is a controversial topic as kidneys do not contain lymphatic tissue [8]. However, some have suggested that PRL can originate from the renal capsule or occur secondary to chronic inflammatory conditions that cause lymphoid infiltration [7].

Diagnosis of DLBL is made by lymph node biopsy. After establishing the diagnosis, whole-body imaging with fluorine-18-fluorodeoxyglucose (18F)-FDG PET/CT is required for disease staging and also to detect nodal and extra-nodal sites [8]. Li et al. reviewed 20 patients with NHL and renal involvement, the most common type of lymphoma was chronic lymphocytic leukemia/small lymphocytic lymphoma (eight patients), followed by DLBL (four patients) [9].

Chemotherapy remains the corner stone of treatment for DLBL. The usual chemotherapy regimen that is used for DLBL is cyclophos­phamide, hydroxydaunorubicin, oncovin and prednisone (CHOP) with or without rituximab protocol [1]. Treatment usually includes 6-8 cycles of the protocol [7]. In a study by Villa et al., patients received at least one cycle of CHOP chemotherapy or similar regimen with curative intent and then received CHOP in combination with rituximab (R-CHOP) [6]. Patients who received rituximab had improved overall survival and progression-free survival. However, this did not appear to reduce rates of CNS relapse. Patients with DLBL and kidney involvement at diagnosis have a poor prognosis in part due to the high incidence of CNS relapses that occur early. Villa et al. concluded that R-CHOP is not sufficient therapy in patients with renal involvement with or without CNS disease and improved diagnostic and treatment modalities are, therefore, necessary for this population [6].

Comparing data for R-CHOP and DA-REPOCH in primary mediastinal B cell lymphoma (PMBCL), the regimen of R-CHOP and radiation therapy had been the standard approach for PMBCL in the United States. However now DA-REPOCH without radiation has become a favored induction regimen for PMBCL [10]. Part of the push for higher intensity regimens for PMBCL has been based on reports of inadequacy with CHOP-based induction chemotherapy. In a retrospective review of 63 patients treated with R-CHOP consolidative radiation, there was a high primary induction failure rate (21%) [11].In PMBCL, the complete remission rate was higher among DA-REPOCH treated patients (84% vs. 70%) and the use of radiation therapy was considerably lower with only 10/76 (13%) DA-REPOCH treated patients receiving consolidative radiation. Overall survival and progression-free survival were similar [10].

Limited data exist on outcomes of patients with DLBL and renal disease who are treated with high-intensity regimes such as DA-REPOCH. Li et al. reviewed 20 patients who had NHL with kidney involvement; DLBL was diagnosed in four patients, three of them died within two years of treatment with CHOP. One patient was treated with steroids and was alive at eight years. Another patient with T/NK cell lymphoma was treated with EPOCH and died in one year [9]. Hanna F et al. reported a patient with two concomitant malignancies, NHL (renal involvement was unilateral in this case) and metastatic prostate adenocarcinoma. The patient was treated with hormonal therapy and six cycles of DA-REPOCH with complete remission [12].

## Conclusions

To the best of our knowledge, this is a rare case of DLBL where the patient presented with bilateral renal involvement and also underwent complete remission after six cycles of DA-REPOCH. It would be worth looking further into outcomes of DLBL patients especially with renal involvement on DA-REPOCH. Multicenter trials are required to demonstrate which of the two chemotherapy regimens (R-CHOP vs. DA-REPOCH) has better progression-free survival in this particular subset of patients.
